# Increasing the Intensity over Time of an Electric-Assist Bike Based on the User and Route: The Bike Becomes the Gym

**DOI:** 10.3390/s18010220

**Published:** 2018-01-14

**Authors:** Daniel H. De La Iglesia, Juan F. De Paz, Gabriel Villarrubia González, Alberto L. Barriuso, Javier Bajo, Juan M. Corchado

**Affiliations:** 1 Computer and Automation Department, University of Salamanca, 37002 Salamanca, Spain; fcofds@usal.es (J.F.D.P.); gvg@usal.es (G.V.G.); albarriuso@usal.es (A.L.B.); corchado@usal.es (J.M.C.); 2 Artificial Intelligence Department, Polytechnic University of Madrid, 28660 Madrid, Spain; jbajo@fi.upm.es

**Keywords:** personalized assistance level, coaching, physical activity, electric bicycles

## Abstract

Nowadays, many citizens have busy days that make finding time for physical activity difficult. Thus, it is important to provide citizens with tools that allow them to introduce physical activity into their lives as part of the day’s routine. This article proposes an app for an electric pedal-assist-system (PAS) bicycle that increases the pedaling intensity so the bicyclist can achieve higher and higher levels of physical activity. The app includes personalized assist levels that have been adapted to the user’s strength/ability and a profile of the route, segmented according to its slopes. Additionally, a social component motivates interaction and competition between users based on a scoring system that shows the level of their performances. To test the training module, a case study in three different European countries lasted four months and included nine people who traveled 551 routes. The electric PAS bicycle with the app that increases intensity of physical activity shows promise for increasing levels of physical activity as a regular part of the day.

## 1. Introduction

Advances in the field of technology and developments in common transport systems have greatly reduced people’s physical activity [1]. In developed countries, the majority of people travel to and from work using motor transport systems, e.g., statistics from 2011 show that, in England and Wales, 85% of the population used motorized transport as their usual commute mode [2]. Nowadays, citizens spend much more time at sedentary activities, such as working in front of the computer. In 2012, data from 66 high and low income countries show that the percentage of adults who spent four or more hours sitting each day was 41.5% [1]. Due to these changes in our lifestyle, the risk of suffering health problems as a result of physical activity is increasingly high [3]. Experts from diverse entities, such as WHO (World Health Organization), recommend an average of 150 min of exercise per week or 30 min daily [4]. Physical activity provides a well-known set of health benefits [5]. Exercise has been proven to reduce the risk of suffering from high blood pressure, stroke and others [4]. It increases cardiorespiratory and muscular fitness, bone health or increased functional health. Moreover, it can help prevent depression [4]. In 2010, the Global Health Observatory (GHO) estimated that the daily physical activity of more than 20% of adults is insufficient [6]. The low exercise, combined with the daily ingestion of fat and calorie rich foods, is leading our society to an obesity epidemic [7].

Nowadays, people who wish to make exercise a part of their daily routine usually go to gyms or sign up for different sports. This commitment implies an economic cost of registration and sports equipment, travel to sports centers, as well as the necessary free time to carry out the activity and a willingness to attend regularly. Performing a team sport (e.g., basketball, football or volleyball) also requires adequate sports facilities, a group of people to carry out the activity and a skill in that sport. For these reasons, many people do not perform physical activity regularly [8,9]. As a result, the daily use of a bicycle in routine trips is an important alternative to the gym or other sports [10].

One of the most widespread ways of fostering active transport among users is by promoting the use of bicycles in the city [11,12]. Biking will not only help people to get fit but will also reduce traffic congestion, environmental contamination, climate change and energetic sustainability [13,14]. The upgrading of infrastructures for cyclists helps provide a positive experience and as a result increases the use of bicycles in the city [15,16]. In recent years, cities have been promoting the use of bicycles through the implementation of bicycle-sharing systems (BSS), in order to allow users to travel short distances by bike [17]. Recent studies have shown that the use of BSS has a positive impact on health [18] and reduces the use of motor vehicles [19,20].

A decisive factor for encouraging the use of bicycles is the development of electric bicycles or e-bikes. An electric bicycle consists of three main elements: Engine, battery and control system. These elements are installed in a conventional pedal assist bicycle, which combines the power exerted by the user with the power supplied by the electric set. In comparison to conventional bicycles, these are power assisted bicycles which can travel greater distances, providing greater mobility and reducing barriers (such as age, physical limitations, steep slopes, and lack of time) [21].

Some people have barriers that make the use of a traditional bike challenging or impossible, however electric bicycles may give them an opportunity to start cycling [22]. Bicycles of this type are essential if an increase in the number of bikers in the city is to be achieved [23]. Electric bicycles can help users get active, especially those who have a sedentary work, can at least do exercise by cycling between home and work [24]. The bicycle industry esteems that the e-bike market will continue to grow. It is estimated that, in the last decade, more than 150 million electric bicycles have been sold worldwide [25]. In 2015, 1.2 million were sold in Europe, and it is estimated that this number will triple in 2022 [25]. The increase of sales is a result of the relatively low cost of these vehicles (generally, less than 1000 euros) and they are increasing in popularity over scooters [26,27]. In addition, the new e-bike BSS systems are increasingly popular among users in comparison to traditional bicycles because they make travel easier and more comfortable when factors such as long travel distance, high temperatures or poor air quality are involved [28]. On the current market, it is possible to find two types of electric bicycles [26]: Throttle electric bikes and Pedal Assist Systems (PAS) e-bikes.

Of these electric bikes, the throttle e-bike [29], offer an acceleration device to the users, similar to the one used on the handlebar of mopeds. With this system, the users can activate and deactivate the assistance of the engine, as well as regulate its intensity. Thus, the user is in total control of the assistance provided by the engine, making the use of bicycles much simpler. The use of pedals and the user’s physical implication is optional, given that the user does not have to put any physical effort into activating the accelerator. Generally, these bicycles are used by expert users who wish to have precise control of the level of power supplied by the engine. In many countries, such as Spain, Finland or the UK, there are strict rules that regulate the users of these bicycles. In Spain, the use of these bicycles on public roads is prohibited and, to be able to use them, the user needs to have liability insurance. 

Assist level electric bicycles or bicycles with a PAS, function differently. Unlike in throttle electric bicycles where, thanks to the acceleration system, the user does not have to exert any physical pressure on the pedals, in PAS bicycles, it is necessary [29]. These e-bikes incorporate a sensor that registers the pedaling velocity of the user and activate the electric engine when pedaling starts. As to the control of the power supplied by the engine, a set of assist levels is employed which will progressively increase or decrease the power provided. A remote control installed on the bike’s handlebar is used to manage assistance levels, it can also be used to interact with any app for smartphones. In this way, the user can freely perform all the operations without removing his hands from the handlebar. The use of these bicycles is also regulated by the law in many European countries. In countries such as Spain and the UK, engine assistance cannot exceed 25 km/h and the electric power of the engine cannot be greater than 250 W [30]. Moreover, the engine has to remain in a resting state when the user stops pedaling. The proposed system is designed for PAS bicycles. Almost all the commercial electric bicycles that we find on the current market are pedal assist.

While electric-bikes lower the levels of physical activity compared with pedaling a regular bike, pedaling a regular bike on flat land for a very short distance is beneficial but not necessarily highly health-inducing. Individuals could go to the gym or engage in sports but that takes time and money. The goal of this work is to increase the levels of physical activity while riding an electric bike, thus allowing the bike to become the gym through an activity that is a routine part of the day. A secondary goal is to achieve an increase in the number of bicyclists, either riding regular bikes or electric-assist bikes. To this end, a personalized pedaling intensity level system for power assisted electric bicycle users has been developed. With this system, users will be able to gradually progress their physical activity, as they travel different routes on their e-bikes. Many studies have shown that riding on a bicycle with constant or incremental velocity in time has significant health benefits [31,32] and contributes to the user’s fitness [33].

## 2. Background

In the current literature, it is possible to find numerous studies that address the use of mobile devices as a means of encouraging physical activity. The authors studied the influence of apps that send motivational messages or messages that provide information on performing an exercise to the users’ mobiles [34,35,36,37,38,39]. The results of these studies show that users’ physical activity improves significantly, e.g., out of the 149 users in the case study [37], the group that used a mobile phone lost per month an average of 0.5 kg more than the control group. In a review, the authors analyze the effectiveness of using a Smartphone for the promotion of daily activity [40]. However, only 6 out of the 13 articles have recorded a change in the behavior of users. Recent studies conducted with patients in medical centers, measured the progress of patients in rehabilitation by counting the number of steps they walked daily, the doctor could then evaluate their progress by comparing these numbers [41,42]. These studies have demonstrated that with the use of a Smartphone and with the supervision of a medical professional, the patients’ daily physical activity could be increased.

Moreover, social environments can also be very effective in encouraging exercise. In other study, the authors questioned whether it would be more effective to encourage physical activity through support among users as opposed to competition [43]. The authors demonstrated that social competition had a greater influence over users than the social support of their friends. Other authors describes a combination of an augmented reality game, such as Pokémon Go [44] with social interaction among users, which succeeded at increasing users’ physical activity [45]. 

The use of video games or active video games (AVGs) as a means of promoting physical activity, has also been widely studied. Authors reviewed 52 articles which were focused on AVG systems in [46]. After the analysis of these studies, the authors pointed to an increased interest in light and moderate activities. However, these systems are not likely to produce any significant changes in sedentary behavior. Only the youngest part of our population could benefit from using AVGs but they are not considered to be an effective tool for increasing daily activity. 

Recent studies, looked for solutions that promote active forms of transport, such as walking or cycling, with the use of Smartphone applications that monitor physical activity [47]. These authors’ goal was to improve active transport and increase daily physical activity when commuting in the city [48]. Electric bicycles were viewed as a new method for promoting daily physical activity [49]. Authors looked at electric bicycles as tools that can benefit the health of elderly people and be a fun and practical way of incorporating exercise into their daily commute [50]. Moreover, authors studied the health benefits of swapping the car for an electric bicycle, especially when used for daily commuting [51]. Other studies analyzed security mechanisms for electric bicycle users, where the user’s heart rate determined the level of assistance that was provided to them [52,53,54]. These mechanisms make it possible for people with health problems to use e-bikes safely.

After a careful analysis of the current literature, no studies were found on the increase of pedaling intensity over time in e-bikes. No relevant research has been conducted in this area. Thus, the proposal made in this study is novel as it is focused specifically on the area of e-bikes and on using them as tools for encouraging physical activity and increasing the intensity of exercise. Therefore, this work proposes a novelty among studies focused on promoting and increasing the intensity of physical activities, specifically for e-bikes. Consequently, this work makes the first reference to this field of study, with the aim of encouraging other researchers to address this problem.

## 3. System Overview 

**Figure 1:**
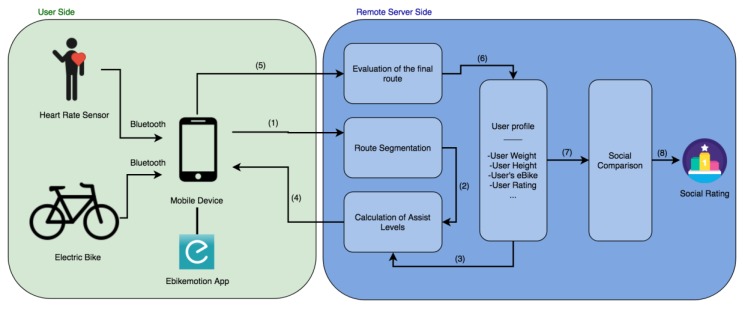
Overall architecture of the system: (**1**) the route selected by the user; (**2**) segmented route; (**3**) user profile data; (**4**) calculated assist levels; (**5**) data collected over the course of the route; (**6**) score obtained on the route; (**7**) comparison of the scores of other users; and (**8**) final social rating.

This section describes the different elements that make up the final system proposed in this work. It is a personalized system whose purpose is to adjust the intensity of exercise for electric bicycle users, in this way facilitating their progress which is marked by the different ability levels. The objective of the system is to promote physical activity among users, with training that is constant and incremental in its intensity. Figure 1 shows the different components that make up the designed system. First, a user of an electric bicycle that is registered in the mobile application, activates the “training” mode and selects the route that they wish to travel. The selected route can be a mountain route or a simple ride, such as the usual route from home to work. Optionally, users can have a Heart Rate sensor which takes their pulse, it allows to measure progress, estimate effort and prevent fatigue. Once the user selects the route they want to travel, it is sent to the remote server which is in charge of managing the data of the platform. When obtained, the server divides the route into segments in order to calculate the power required to travel the route. This is done by establishing the assist level for each of the segments; this level is calculated by considering the user’s physical characteristics (height and weight), his ability level (beginner, intermediate, advanced), the characteristics of the electric bicycle (power, battery, weight) and the profile of the route (slope and distance). The objective of calculating assist levels is to prevent excessive variations in velocity over the whole route, by combining the power supplied by the engine with the power provided by the user. The power provided by the user will increase gradually, with each of the exercises that he completes on the platform. Time will not be the only factor taken into account when calculating the difficulty of the travelled route, the slopes found on that route will also be considered. In this way, it will be possible to compare the different routes more effectively. 

The resulting assist levels are sent to the user’s mobile application, together with the waypoints indicating the beginning of each segment. Over the course of the ride, the assist levels will change automatically when the user reaches the different waypoints. Once the route is completed, the application will send the data registered over the course of the route to the server, which will proceed with their analysis. After the evaluation of the results, the system moves on to calculating the score obtained by the user in the route he completed. The scores obtained in different routes accumulate to a total and the general rating of the user is obtained. These data are necessary for evaluating the user’s progress and for calculating the assist levels of future routes. 

Finally, the system has an interactive component based on the development social competition. This element allows users to view their progress in comparison to others, to suggest improvements and routes based on their profile. It also allows motivating them through a series of general ratings and the ratings made by friends who use the application.

### 3.1. Electric Bike and Sensor Data

#### 3.1.1. Assist Levels

As described in the previous paragraph, PAS bicycles increase or decrease the power supplied by the engine with a set of assist levels. Not all electric bicycle manufacturers configure assist levels in the same way. However, in the majority of cases, assist levels oscillate between 5 and 10 levels. There are also bicycles that have a lower power engine which only has a total of three assist levels. However, independently of the assist levels available in bicycles, their functioning is similar in the majority of cases. Each of the assist levels provides an incremental percentage of power. Higher levels provide greater power than lower levels. 

**Figure 2:**
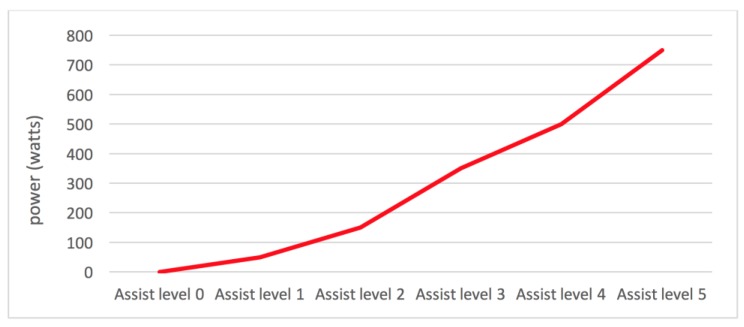
Assist levels and power in an e-bike of 750 w.

Figure 2 shows a graph of the power supplied by an electric bicycle with an engine of 750 W and a total of five assist levels. As it can be observed, the first assist levels provide less power, what means lower velocity, while the highest level provides the maximum power of the engine. In some bicycle models, it is possible to configure engine settings associated with assist levels. In this way, an advanced user could configure the established power profile, so that it suits his needs. The relation between watts and assist levels is obtained directly from the mobile application, since it is possible to monitor the intensity of the battery current at the different levels. Thanks to this possibility, the system is suitable for different types of batteries and engines with no previous configurations.

**Figure 3:**
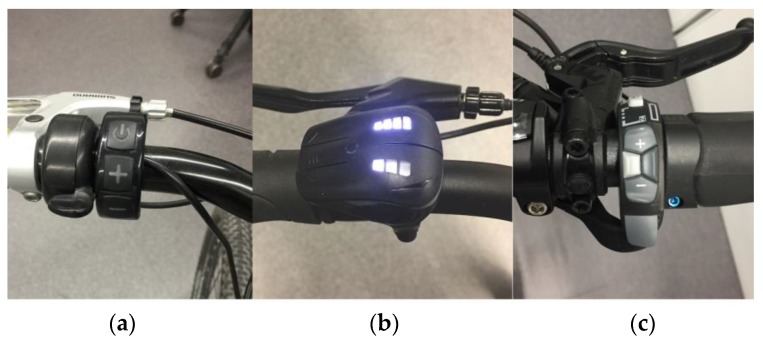
Example of three assist level commercial controllers: (**a**) Bafang controller; (**b**) *ebikemotion* controller; and (**c**) BionX controller.

When controlling assistance, it is necessary to have a device connected to the bicycle’s control system, capable of increasing and decreasing the power. Commercial bicycles have a remote control installed on their handlebar, through which the user can control the behavior of the electric system. Figure 3 shows three different models of remote controls for electric bicycles. Generally, these remote controls, besides an on/off button, also have two additional buttons: one for increasing the assist level and another for decreasing it. They also incorporate Bluetooth wireless communication technology. Some models, such as the iwok model (Figure 3b), incorporate auxiliary buttons, which make it possible to interact with the *ebikemotion* mobile application. 

The remote control is not the only control interface for the assist levels of an electric bicycle. Manufacturers incorporate a control interface in their communication protocols through commands sent by a third party, such as a mobile application. Thanks to this interface, the assistance of an electric bicycle can change automatically and it is not necessary for a user to intervene. This is a fundamental element in this work; as the user travels a route in the “training” mode, the system will change the assist levels in the e-bike in a dynamic and independent manner, by means of the application.

#### 3.1.2. Heart Rate Sensor

The system measures the heart rate by means of an external wireless heart rate sensor connected to the app by Bluetooth. Any of the current commercial sensors with Bluetooth 4.0 is compatible and can be used in the system. There are two reasons for which in the proposed system the user’s heart rate is measured by an external sensor. On the one hand, to register the user’s improvement along the different exercises he does, which is key for establishing the level of progress and physical development. Users who are not physically fit have a greater number of ppm (pulsations per minute) than users who are used to exercise. The continuous evaluation of changes in the heart rate, while the user performs physical activities, is an important indicator of the user’s progress, as reported previously in different works [55].

Similarly, the heart rate registered during a physical activity, such as cycling, provides a measure of the athlete’s effort. When calculating the training thresholds, the time the user spent exercising at each of the training zones has to be considered together with the maximum heart rate. This value is calculated based on Equation (1), which has been previously described in the literature [56]. This is the most accepted way of calculating the heart rate even though it has a significant margin of error, so it should be considered as an approximate value and in no case as a precise value.

(1) $${\mathrm{HR}}_{\max\;}=205.8-0.685\cdot \left(\mathrm{age}\right)$$

Table 1, is a general list of the four main training zones for an athlete during physical exercise, as described in [57]. The designed system can calculate the percentage of time that a user spent training in each of these zones, for each of the routes he travelled; this measures the quality of exercise. Zone 1 is considered as exercise that is safe for the heart and is recommended to users who are only beginning to introduce physical activity into their daily routine and who are not physically fit. Zone 3 is considered the anaerobic threshold and it is a turning point in the improvement of capabilities, from here a decrease in performance can be observed. Lastly, training zone 4, can only be maintained during a few seconds and can only be achieved by users with a high level of physical training. 

The use of a heart rate sensor is not only important for measuring physical progress. In the designed system, the monitoring of the user’s pulse is also seen as a security measure that helps to avoid and prevent fatigue. When the system detects the user’s pulse to be very high, over the maximum threshold, the assistance system increases the power of the engine automatically. In this way, the user is helped in his exercise and their heart rate is reduced. This threshold can be established manually by the user on the mobile application or it can be calculated automatically with Equation (1). 

#### 3.1.3. *ebikemotion* App

**Figure 4:**
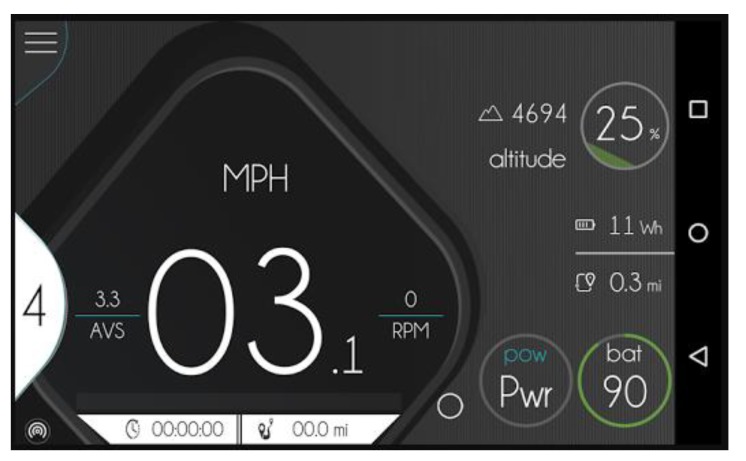
Screenshot of *ebikemotion* app.

The *ebikemotion* project has been co-developed by the University of Salamanca and the company StageMotion [58]. The *ebikemotion* application for mobile devices [59] is central to the system developed in this article. This application, is compatible with more than 20 electric bicycle brands on the market and it has more than 5000 users from all over the world. This application visualizes all the values of the electric bicycle in real-time (battery level, assist level, velocity, altitude etc.) as can be seen in Figure 4. This application is free and is available in the two main mobile operating systems, Android and iOS. The application was launched in the middle of 2016 and it is possible to use it with electric bicycles as well as with traditional bicycles for recording routes via GPS.

As part of this work, a “training” module has been designed for this application. This application will be in charge of registering the values of the different routes travelled by the user in the “training” mode and of automatically changing the assist levels on the basis of the parameters calculated by the server. The application is linked to the e-bike by Bluetooth wireless technology. Some e-bike models obtain their Bluetooth connection through the remote control while in other models this technology is incorporated in the casing of the battery of the e-bike.

### 3.2. Route Segmentation

The routes travelled by the users through the exercise plan have a high number of GPS localizations. First, it is necessary to identify each of the segments that make up a given route. To this end, the route is segmented into independent sections of different lengths that are connected to each other. The aim of performing this segmentation is to be able to make an individual analysis of each section; reducing its complexity and establishing the assist level required for that route. As described previously, electric bicycles are operated through a series of assist levels, where each one corresponds to a particular amount of power that is provided by the engine. The higher the assist level, the greater the power generated by the engine. Thus, the higher is the assist level, the lesser is the effort of the user. 

**Figure 5:**
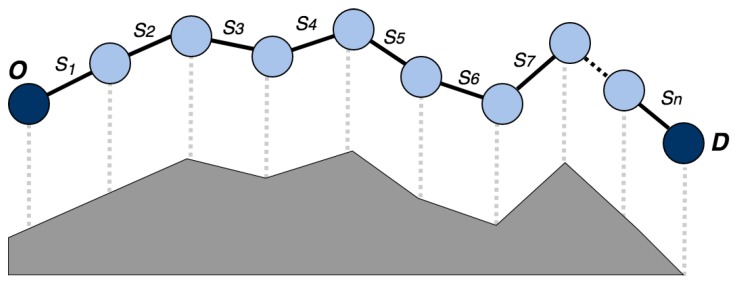
Example of route segmentation. The bottom side and the upper side of the route is segmented according to its profile.

The second criterion for making this division is based on the difference of slopes at the different points of the route. Slopes are obtained for each of the GPS points *p*, therefore it is possible to group the adjacent route points which have similar magnitude of slope $g_{i}$, in the same segment $S_{i}$. The segments obtained can have different lengths $l_{i}$. Thus, a route with origin O and destination **D** is divided into a series of i=1, …, N segments $S_{i}$, which are connected one to another, as shown in Figure 5. 

By grouping the points *p* according to the magnitude of the slope, the profile of the route is reconstructed with the set of segments $S_{i}$, as can be seen in Figure 5. Thanks to this grouping, it is possible to establish a single assist level for each segment; this level will be adapted to its slope. When calculating the starting and ending waypoints of a segment, not only the magnitudes of these points should be considered but also the magnitudes of the adjacent points.

The algorithm used to perform this segmentation is based on the previous work of the same authors [60]. In the segmentation algorithm, does not only consider whether the slope is positive or negative, its magnitude is also considered. In this way, within segments with slopes of the same signs, different segments can be grouped according to their magnitude. As for segments of 0% slope, they are considered to be positive or negative, according to the adjacent slopes. 

### 3.3. Calculation of Assist Level

When the system calculates the assist level for each segment, two elements are taken into account. On the one hand, the user’s ability level (calculated with the scores obtained on the routes they travelled previously in the “training” mode). On the other hand, the average slope of the segment within the route. The objective is to apportion the power required, according to the level of assist for each of the segments. The power provided by the engine must be complemented with the power derived from the user’s physical effort. Thus, the sum of the power to be supplied by the engine and the power that is to be provided by the user in each of the segments, is the total power required to travel the route, as shown in Equation (2).

(2) $$\overset{n}{\underset{i=0}{\sum }}(p_{g}+p_{u})=Total\;power\;to\;travel\;a\;route$$

where $p_{g}$ represents the power supplied by the engine and $p_{u}$ the power supplied by the user, in each segment n of a route.

To calculate the power required to cycle a bike route, it is necessary to apply Equation (3) as described in [61].

(3) $$P=k_{r}Ms+k_{a}Asv^{2}d+giMs$$

where

- P: Represents the total power required to cycle a route, in watts.
- $k_{r}$: Rolling resistance coefficient, is a constant (0.005).
- M: Mass of the bike and the cyclist.
- s: Speed of the bike on the road.
- $k_{a}$: Wind resistance coefficient, is a constant (0.5).
- A: The frontal area of the bike and cyclist.
- v: Speed of the bike through the air (bike speed +headwind or −tailwind).
- d: Air density, is a constant (1.226 kg/m^3^).
- g: Gravitational constant (9.8 m/s^2^).
- i: Gradient (slope).

Equation (3) can be divided into three different parts. In the first part ($k_{r}Ms$) calculates the power necessary to overcome the resistance produced by the friction between the wheel and the ground. For this part, it is necessary to add the power required to overcome wind resistance ($k_{a}Asv^{2}d$) and finally the power required to overcome the resistance of the slope of the road (giMs).

To calculate the total mass (sum of the mass of the user and the mass of the bicycle), the user’s profile with their data is accessed. The weight of the bicycle is also known since the system registers the bicycle that the user is using to cycle a route. Likewise, knowing the user’s height and the size of the bicycle, makes it possible to obtain the user’s frontal area.

For the system designed in this work, speed is considered to be a key factor. From the user’s overall score (which is the sum of the points obtained at previous routes), the average speed that is to be maintained constant by the user during the completion of a new route is determined. The goal of the “training” mode is to maintain a constant average speed throughout the route, regardless of its profile, and to increase that speed progressively at new routes. In this way, the user gradually observes progress in his training, as the average speed at the routes they cycle is increased and the power supplied by the engine is reduced. Table 2 shows the ability levels established in the system and the score necessary to reach each one of them. The table also indicates the average speed at each of the levels which is used to calculate the power. These power estimates are defined, in ideal conditions, for a person of 75 kg and a bicycle of 8 kg (a total of 83 kg).

Each route is divided into a set of segments, as shown in Figure 5 and Equation (4).

(4) $$r=\left\{S_{1},‥,S_{n}\right\}$$

The slope of each segment within a route is calculated, thus obtaining a set of values for each of the segments (Equation (5)).

(5) $$I=\left\{h_{1},‥,h_{n}\right\}$$

For each segment *S_i_*, the automatic assist level is calculated. First, the power that is to be generated by the user is established. The power *p* is kept constant for each of the intervals and the speed of the user is determined using the formula indicated in Equation (3). The speed calculated for each interval is shown in Equation (6).

(6) $$V=\left\{v_{1},‥,v_{n}\right\}$$

At this point, the tentative speed *v*’ that is to be maintained is considered and the power that should be generated to maintain that speed *v*’ is calculated for each interval (Equation (7)).

(7) $$P=\left\{p_{1},‥,p_{n}\right\}$$

The power supplied by the battery will be the difference between the power required to maintain the speed constant at each interval and the *p* value maintained by the user (Equation (8)).

(8) $$P\prime =\left\{p_{1}^{\prime }=p_{1}-p,‥,p_{n}^{\prime }=p_{n}-p\right\}$$

Once the $p_{i}^{\prime }$ values are obtained, the level of assist is calculated. Each assist level *r* is associated with a power $p_{r}^{l}$, whereby the possible power values are as given in Equation (9).

(9) $$p^{l}=\left\{o,p_{1}^{l},‥,p_{r}^{l}\right\}$$

Based on the power values defined in Equation (9), the power intervals are established Equation (10).

(10) $$I=\left\{\left[0,\frac{0+p_{1}^{l}}{2}\right),\left[\frac{p_{1}^{l}+p_{2}^{l}}{2},\frac{p_{2}^{l}+p_{3}^{l}}{2}\right),\ldots ,\left[\frac{p_{r-1}^{l}+p_{r}^{l}}{2},p_{r}^{l}\right)\right\}$$

Therefore, the assistance for segment *q* defined as $p_{q}^{e}$, is represented by Equation (11).

(11) $$p_{q}^{e}=p_{j}^{l}/\left[\frac{p_{j-1}^{l}+p_{j}^{l}}{2},\frac{p_{j}^{l}+p_{j+1}^{l}}{2}\right)$$

**Figure 6:**
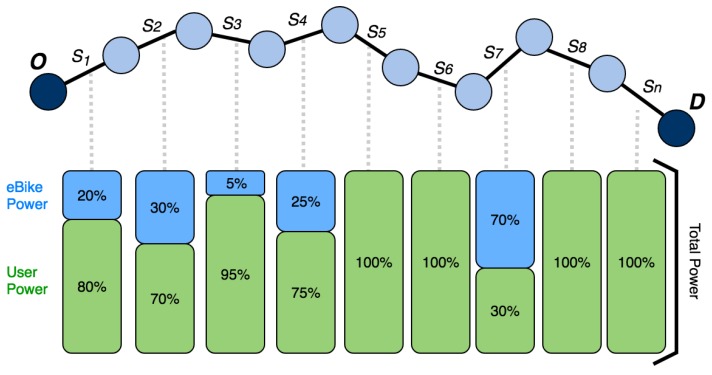
Example of how power is distributed for each of the segments of a route.

As shown in Figure 6, after establishing the assist levels for each segment, the amount of power that the user must provide and the power that the bicycle must supply is determined. The total sum of the powers must be equal to the power required to travel the segment at a constant speed. 

### 3.4. Evaluation of the Final Route

An important aspect is the evaluation of the user’s performance on the routes cycled. However, to be able to make a fair evaluation of these routes it must be taken into account that the profile of each route is different. Thus, a procedure has been designed to make a fair comparison. The power generated by the user over the course of the route is the aspect that is considered in the evaluation; as it can be seen in Equation (3), the calculation of this power depends on a number of factors. The power generated by the user is defined in Equation (12).

(12) $$p_{u}=\overset{n}{\underset{i=1}{\sum }}\left(p_{i}-p_{i}^{e}\right)$$

Once the power generated by the user has been calculated, the system determines the final score that is to be awarded for the route represented by $s_{r}$. This score will accrue to the one already obtained by the user. The score is therefore calculated as Equation (13):

(13) $$s_{r}=\{\begin{array}{cc} p_{u} & p_{u}<600\\ 600 & eoc \end{array}$$

The value of 600 W is considered as the energy required to maintain an average speed of 50 km/h for one hour on a flat surface, considering a user of 75 kg and a bicycle of 8 kg and with average parameters for the coefficients of friction, resistance etc. Considering the records registered by International Cyclist Union [62], the 600 W value is the maximum limit for a person. 

### 3.5. Social Competition for the Promotion of Physical Activity

The objective of the proposed system is to promote users’ physical activity through the cycling of routes on electric bicycles. Thanks to social interaction through the application, users are motivated to compete with each other by increasing their physical activity. As described previously, the application implements a social component in order to provide users with a comparative of their progress. The system generates three different rankings on the application; general ranking by area (includes the scores of users in the same location) which shows 10 users with higher scoring. Another ranking with users who have similar profiles (age, gender, routes cycled, the number of routes cycled per week). Finally, a ranking with the user’s friends on Facebook. The *ebikemotion* application has native support for Facebook, making it simple for users to find friends on the social network through the application. Eighty-six percent of current *ebikemotion* users have their Facebook account linked to the application, this shows that social interaction among users is high. 

## 4. Case Study

To validate the proposed system, a case study was carried out with real participants using the platform and the application for mobile devices called *ebikemotion*. The testing took place over four months between March and June of 2017 and included nine participants who travelled a total of 551 routes. Out of the nine case study participants, seven were located in Spain (five in Salamanca and two in Palencia), one in Italy (Volverra) and another in Switzerland (Corseaux). All of them are members of the team that worked on developing the software and hardware of the *ebikemotion* platform and on its testing. None of the participants presented any notable health problems and they were not remunerated for their collaboration in the study. All participants wore a heart rate sensor that was compatible with the app. After completing the study, the users made a personal evaluation of their experience in the study.

Before starting the study, the participants’ physical fitness was determined to make two groups of users: Users with high physical activity and users with low physical activity. Users with high physical activity are those who practice sports for more than 6 h a week, including cycling. Users who have low physical activity are those who do exercise for less than 3 h a week and do not use the bicycle. The objective of distinguishing these two groups is to be able to compare the progress of the most active users with that of less active users. Table 3 defines the characteristics of the nine users participating in the study. As can be seen, Users 1, 2, 3, 5, 8 have a high activity during the week, exceeding 9 h of exercise per week, while Users 6, 7, 4, 9 have very little physical activity during the week. 

For the purposes of this case study, all participants had an electric bicycle at their disposal. All bicycles were equipped with the PAS system and were compatible with the *ebikemotion* application. The bicycle of User 1 was road type while the rest of the users’ bikes were MTB type or city bikes. The power of the engines of the different bicycles oscillated depending on their model, ranging in their maximum power from 250 W to 750 W.

## 5. Results and Discussion

### 5.1. Route Analysis

**Figure 7:**
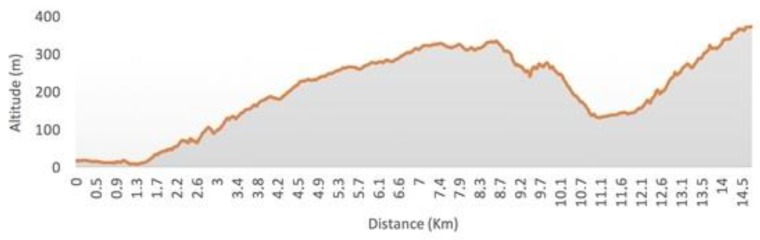
Profile (in altitude) of the analyzed route.

**Figure 8:**
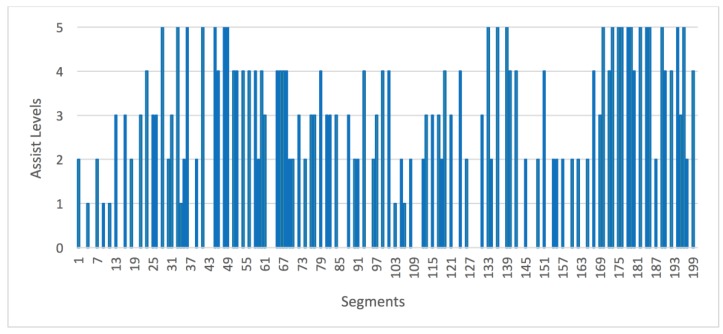
Levels of assistance calculated for each segment.

To illustrate how the route analysis system described in the work functions, a detailed analysis of one of the routes cycled by one of the users was carried out. It is a 14.5 km route in the city of Corseaux (Switzerland) whose altitude profile is shown in Figure 7. The route was made with a 750 W electric bike with a configuration of six levels of assist, as shown in Figure 2. First, the route was processed using the previously described algorithm to divide it into segments. After this analysis, the system determined that the route was composed of a total of 200 segments. Next, the system calculated the power that the user must provide and the power that the engine must supply to perform the exercise. As detailed in Section 3.3, in this calculation, it is necessary to consider the slope of each segment and the user’s ability level in the system. In this case, the user had ability level 4, so the system determined their average speed to be 19 km/h over the course of the route. Based on these data, levels of assist are established for each of the segments. The resulting set of assist levels is shown in Figure 8. Finally, these assist levels are sent to the mobile application and the user begins the route.

**Figure 9:**
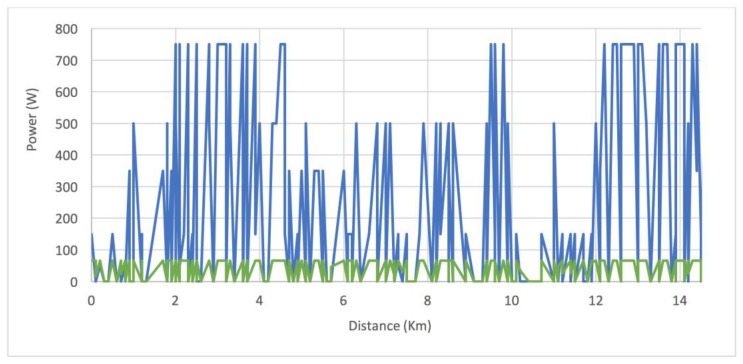
Power apportioned to each of the segments of the analyzed route. The power provided by the user (in blue) and the power provided by the electric engine (in green).

Once the route is completed, the data collected over the course of the route are sent to the central server for analysis. In Figure 9, we can see the actual power that was provided by the engine (in blue) and the power provided by the user (in green). First, Figure 8 shows how the amount of power is adjusted to the slopes of the route. A greater amount of power has been provided in the segment from 2 to 4 km, due to the inclination of that part of the route. The same happened in the segment from 9 to 10 km and in the final segment of the route, from 12 to 14.5 km. On the contrary, at the segment starting at 10 km and finishing at 12 km, the power provided is lower since the slope of this part of the route is descending. It is also possible to observe how the user’s speed is kept as constant as possible on the slopes, although if the selected speed is very high, the power of the engine does not allow the speed to be maintained. With this system, the user’s physical effort is not affected by the profile of the route. Regardless of the profile of the route, speed is kept as constant as possible; this helps prevent fatigue and makes exercise healthier. In addition, a heart rate monitor is employed as a safety measure which allows to increase the level of assistance automatically when the threshold of pulsations per minute is exceeded, as indicated in Section 3.1.2.

The total watt hours consumed when the route was completed were 240 Wh: 210 Wh were provided by the electric assistance system and 30 Wh were provided by the user. Therefore, the user was awarded a final score of 30 points upon the completion of the route.

### 5.2. Results Overview

**Figure 10:**
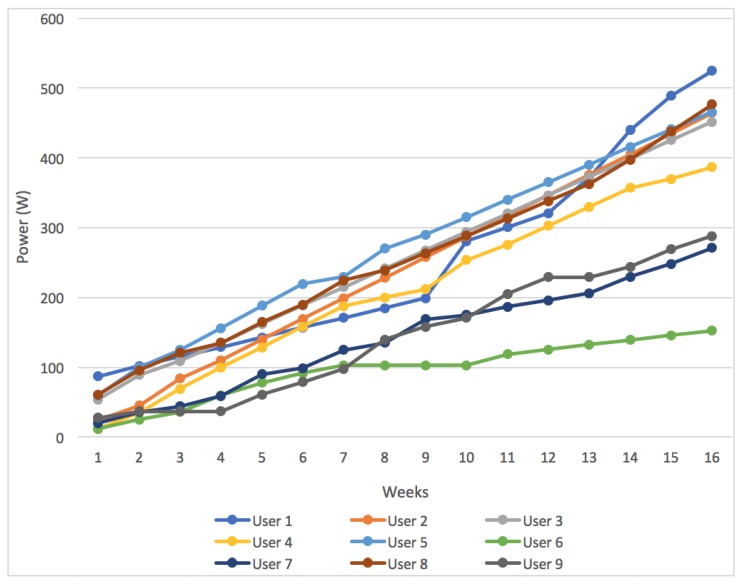
Results obtained by the nine case study participants.

The results obtained at the end of the four-month case study are discussed below to show the progress of the participants. Figure 10 shows the results obtained by the participants and it illustrates the powers each of the users reached to, over the 16 weeks (four months). 

As can be seen, most users have exceeded 300 W, which means that they reached ability level 7 in the system. These users could cycle flat routes with an average speed of 23 km/h, which implies a high level of physical activity. Only three users (6, 7, and 9) were below level 7 in the system, since they did not exceed the 300 Wh. Considering the data analyzed in Table 3, these users do the least amount of exercise per week. After participating in this case study, the three least active users, have managed to do an average of between 4 and 6 h of physical activity a week. The most active users, such as Users 1 and 8, have increased their activity exponentially, surpassing the number of hours that they normally spent cycling. 

**Figure 11:**
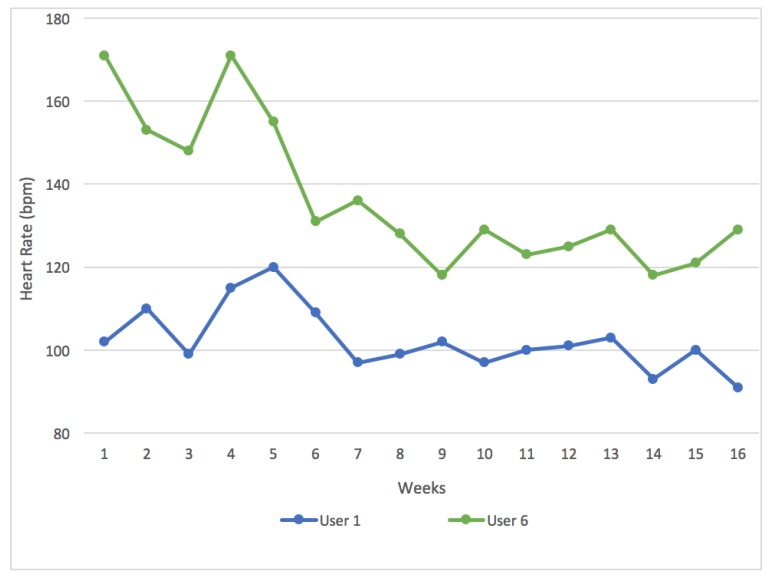
The evolution of the user’s heart rate during exercise, over the course of the case study.

After analyzing the data provided by the heart rate sensor, it was found that the progress of users who exercised regularly throughout the week was less evident than the progress of those who did little exercise. These data are influenced by factors such as the fitness of the user, the duration of the routes and their difficulty. Users who traveled longer routes with higher slopes, made a greater physical effort and therefore their average heart rate had been affected. However, we can look at the data of the two most representative users of the two groups, users who spent a considerable amount of hours exercising each week and those who did little exercise over the week. Figure 11 compares the mean evolution of the heart rate of these two users over the 16 weeks during which the study had been conducted. In the less active group, User 6 did an average of 1 h of exercise each week. In the first weeks, the user’s heart rate was high, however it gradually lowered over the next weeks. On the other hand, the progress of User 1 who did 13 h of physical activity weekly, is not as pronounced. This is because active users’ heart rate value tends to stabilize once they have reached a stable level of fitness.

**Figure 12:**
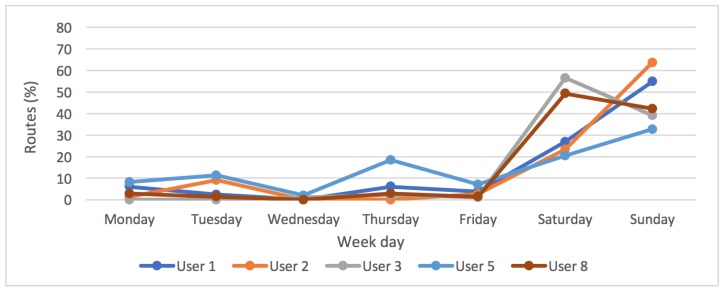
Distribution of the routes cycled by users from the first group over the week.

If we look closely at the percentage of routes performed according to the days of the week, we can clearly see that there are two groups. On the one hand, users whose cycling activity is high and constant on the weekends. In Figure 12, it can be read that users in this first group did their activities between 30% and 65% on Saturday and Sunday. While the activities carried out between Monday and Friday do not exceed 15% on average. This kind of users (Users 1, 2, 3, 5 and 8) have improved their average speed significantly over the 16 weeks.

**Figure 13:**
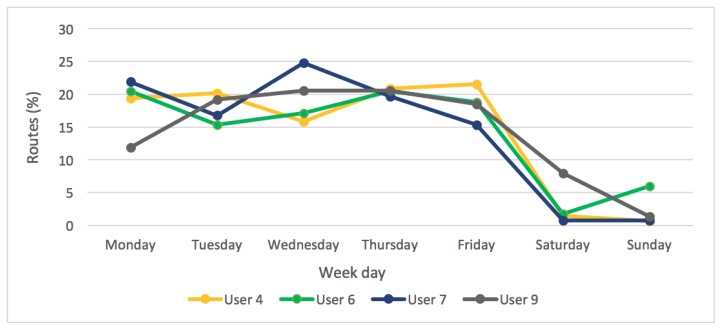
Distribution of the routes cycled by users from the second group over the week.

Figure 13 shows the data of the rest of users, the second user group. These users perform a greater amount of activities over the week than during weekends. In this case, on average, 85% of activities were done between Monday and Friday. This is because the users used their bicycles to commute daily in the city. Cyclists such as User 6 progressed in the number of hours they cycled over the week, from 1 h of exercise a week to an average of 5 h per week, thanks to the support offered by the electrical assistance system.

**Figure 14:**
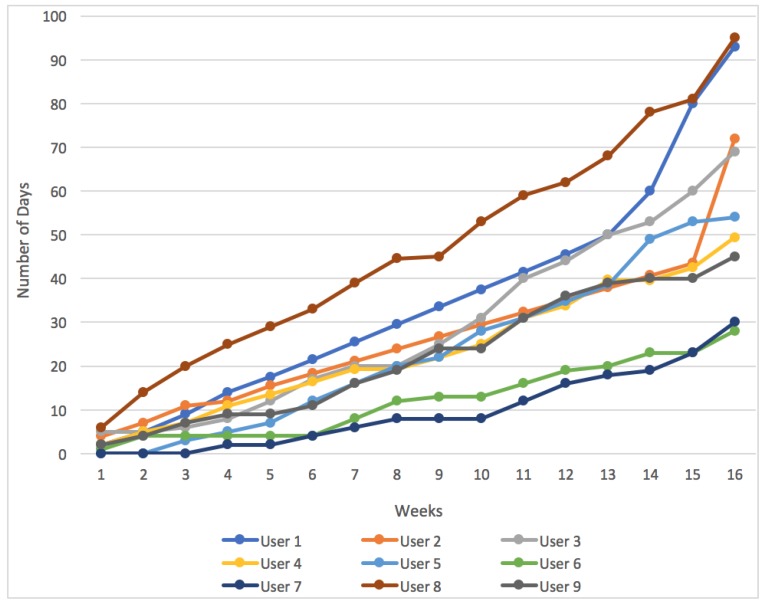
Number of days over the duration of the study in which users consulted social statistics.

The web application took record of the days that users checked social statistics on their accounts. The statistics provided by the web application can be seen in Figure 14. In general, all users monitored their performance on the platform. The number of times users accessed these statistics was low in the beginning of the study, however it started increasing with time, especially in the final weeks. Specifically, the activity of Users 1, 2, 3, 4, 8 and 9 increased significantly towards the end of the study and Users 3, 4, 7 and 8 were increasing the number of their cycling activities because they were competing with other users for a higher score. This increase in activity can be seen in Figure 10 in the last weeks of the study.

**Figure 15:**
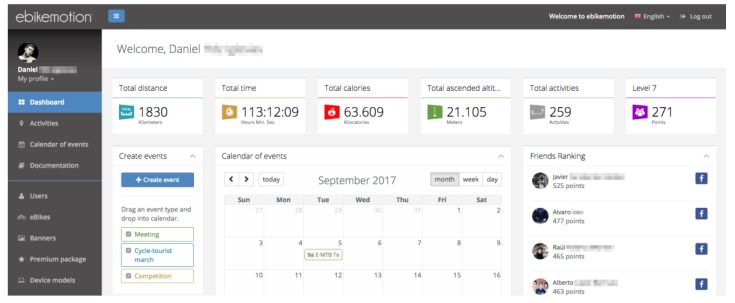
Screenshot of the visualization of results on the *ebikemotion* web application. The names of the users have been erased for privacy reasons.

Finally, Figure 15 shows a screenshot of the *ebikemotion* web application, where the results of the system can be visualized. On the bottom right-hand side, the rankings of friends who are also users can be viewed. In addition, it is possible to visualize other parameters such as the routes cycled, the calories burned or the scores obtained and the user’s ability level.

### 5.3. Case Study Limitations

It should be noted that the case study participants were located in four different cities, in three different European countries. In this study, it is not possible to see concrete behavior patterns in the evolution of the users from different countries, however it should be kept in mind that the cycling culture varies from country to country. Factors such as climate, infrastructures or terrain orography have an influence on the mode of transport chosen by citizens. It is also important to note that users have not travelled the same routes. Each user travelled different routes with different characteristics. For this reason, it is not possible to directly compare routes with others. The value of the heart rate sensor depends on the physical activities performed by the users. Those users who have performed activities that demand more physical activity, have higher HR values than users with simpler routes.

## 6. Conclusions

In this work, a personalized intensity level system for the users of assisted electric bicycles has been designed and implemented. The designed system establishes different assist levels in a personalized way, considering the profile of the route, the power required and the user’s ability level. As the user travels new routes, the system awards them with higher scores. The higher the score, the greater the average speed on the routes cycled by the user and the greater the amount of power that the user needs to generate. Thanks to the progressive increase in speed, the user gradually does more physical exercise, improving and increasing their fitness. Therefore, it is possible to replace the gym with the use of the electric bicycle for daily commutes saving economic and time costs. This is an important finding of this work.

The innovative component presented in this work is the personalized calculation of exercise for electric bicycle users. Thanks to this system, the user will be able to cycle the routes according to their physical state and ability level. As the user moves up the designed ability levels, the cycling difficulty increases. As demonstrated in Section 5.1, where a route cycled by one of the users has been analyzed, the performance of the designed system is satisfactory. It can segment the route according to its slopes and establish the power that is to be provided by the user, according to its characteristics. The proposed system also evaluates the data collected along the route that had been cycled. This study also demonstrated that the amount of hours the nine case study participants spent on physical activities in a week increased over the four months. This improvement was achieved for both users who were physically fit and those that were not. In general, all users said they were satisfied with their results upon the completion of the 16 weeks of testing. The users whose previous average activity was low (between 0 and 2 h a week) reported that the combination of the e-bike and the training module had helped them increase the amount if exercise they did weekly. The users who were used to regular exercise said that the scoring system and social competition had motivated them to further increase the number of hours they dedicated to exercise weekly.

Thanks to the novel system with assist levels, the more advanced users could progress quickly while the users who were less prepared made a gradual and constant improvement over the four months. The case study participants were located in four different cities, in three different European countries. In future work, a case study will be conducted with users from different parts of the world, whose areas will be more heterogeneous. In the future, we would also like to validate the feasibility of the system in terms of its suitability for people of different ages. To this end, we will conduct a case study that will divide participants into different age groups, such as young people, adults and the elderly.

## Figures and Tables

**Table 1:** Heart Rate training zones.

Target Zone	Intensity% of HR_max_ (bpm)	Training Benefit
Zone 1: Light	50–60%	Increases overall health and metabolism
Zone 2: Moderate	70–80%	Improves aerobic fitness
Zone 3: Hard	80–90%	Increases maximum performance capacity
Zone 4: Maximum	90–100%	Increases maximum sprint race speed

**Table 2:** Ability levels, average speed and the score required for each level.

Level	Average Speed	Total Points	Power in 0% Slope
Level 1	15 km/h	[0, 50]	33.19 Watts
Level 2	17 km/h	[51, 100]	42.35 Watts
Level 3	18 km/h	[101, 150]	4757 Watts
Level 4	19 km/h	[151, 200]	53.27 Watts
Level 5	20 km/h	[201, 250]	59.46 Watts
Level 6	21 km/h	[251, 300]	66.17 Watts
Level 7	23 km/h	[301, 350]	81.27 Watts
Level 8	25 km/h	[351, 400]	98.76 Watts
Level 9	26 km/h	[401, 500]	108.47 Watts
Level 10	28 km/h	[501, 600]	129.95 Watts

**Table 3:** Set of users for the case of study.

User	Sex	Age	Hours of Activity/Week
User 1	Male	33	13 h
User 2	Male	29	10 h
User 3	Male	32	9 h
User 4	Female	26	1 h
User 5	Male	42	10.5 h
User 6	Female	31	1 h
User 7	Male	28	0 h
User 8	Male	30	0 h
User 9	Female	27	1 h
